# Onchocerciasis in the Upper Imo River Basin, Nigeria: Prevalence and Comparative Study of Waist and Shoulder Snips from Mesoendemic Communities

**Published:** 2010-06

**Authors:** EC Uttah

**Affiliations:** Department of Biological Sciences, Faculty of Science, Cross River University of Technology, Calabar, Nigeria

**Keywords:** Onchocerciasis, *Onchocerca volvulus*, Prevalence, Corneo-Scleral Punch, Nigeria

## Abstract

**Background:**

Onchocerciasis is endemic in the Imo River Basin, Nigeria. This study was aimed at assessing the prevalence and intensity of microfilaria of *Onchocerca volvulus* in the area.

**Methods:**

A cross-sectional study was carried out in the Okigwe Local Government Area, Imo State, Nigeria. Two skin snips (one from the waist and another from the shoulder) were taken from 1024 individuals examined. The survey coverage was high (91.8% of the study population). An individual was considered mf positive if either of the waist or shoulder snips or both were mf positive. The SPSS for Windows package was used for entering and analysis of data.

**Results:**

Thirty-seven percentage of those examined was positive for *Onchocerca volvulus* microfilariae (39.2% of males and 34.9% of females). The mf prevalence increased steadily with increasing age to reach 70.4% in the oldest age group. The overall mf Geometric Mean Intensity among mf positive individuals was 16 mf/skin snip and was significantly higher among males (18 mf/skin snip) than females (14 mf/skin snip) (p<0.01). A scatter plot of microfilariae numbers in snips from the waist against numbers in snips from the shoulder of the same individuals, showed close correlation (Pearson's correlation coefficient=+0.90; p<0.01), and those with mf intensities below 10 mf/snip had a more scattering tendency away from the regression line than those with higher mf intensities.

**Conclusion:**

Onchocerciasis is a public health concern in the area. Perhaps, 10 mf/snip is critical intensity threshold for reliable sampling using corneo-scleral punch.

## Introduction

Onchocerciasis is a severe and debilitating parasitic infection of global concern. Its prevalence and the magnitude of associated social and economic effects vary widely in different geographical areas where the disease occurs (1, 2). About 90 million people are at risk of which 17.6 million are infected, including 326,000 people who have gone blind, in 34 countries of the world (3). In Africa alone, home to over 96% of all global cases, it has been reported in 26 countries (1).

Onchocerciasis is perhaps the most studied filarial infection in Nigeria. The provisional estimates had suggested that 7–10 million Nigerians are infected with *Onchocerca volvulus*, approximately 40 million are at risk of the disease (4), and 120,000 cases of onchocerciasis-related blindness (1), with many thousands suffering from disabling complications of the disease (5). New foci of onchocerciasis are still being discovered and therefore its distribution could be far more extensive than has been earlier assumed (6).

In southeastern Nigeria, there are pockets of endemic foci, as shown by some reported studies (7–13) although there is gross underreporting of the scourge. Arguably, the most significant area in this sub-region as far as onchocerciasis is concerned, is the hilly and undulating Udi-Enugu-Okigwe axis from where some rivers or their tributaries, supporting black fly vector breeding, have their origin. These include rivers such as Oji, Ajali, Mamu, Adada, and Imo (the biggest of them). Unfortunately, studies in this sub-region have been largely cross-sectional. There has not been any comprehensive study on all aspects of the infection: parasitological, clinical, and epidemiological.

This report is the first emanating from this holistic study on meso-endemic onchocerciasis. It is aimed at assessing the prevalence and intensity of microfilariae of *O. volvulus,* and to compare the microfilariae and mf intensity of skin snips from the waist and shoulders of same individuals with a view to ascertaining the critical threshold for reliable sampling using the corneo-scleral punch.

## Materials and Methods

### Study area and study population

The study was conducted in 2005 in two neighboring high altitude communities of Umuowaibu 1 and Ndiorji, in Okigwe Local Government area of Imo State, Nigeria. The two communities with a combined population of 1,116 at the time of this study are socio-culturally similar, both inhabited by Ndiigbo, the majority tribe in southern, Nigeria. A familial settlement pattern was evident in the area with houses arranged in family clusters. A total of 381 houses were recorded in the two communities, 216 in Umuowaibu1 and 165 in Ndiorji, giving an overall average of three persons per house.

The area is hilly with characteristic undulating plains. There are a total of seven streams and three rivers in addition to the Imo River. There are two distinct seasons, the dry season (November – May), and the rainy season (June – October). According to data from the Imo State Meteorological Service, the annual rainfall in the area averages 2,840 mm per annum, with most of the rainfall occurring in the months of June through October. The mean relative humidity was between 68.7% and 74.4%. Farming is the main occupation, however, those who are engaged in other occupations engage in subsistence farming.

### Preparation for the study

Local Government Area (LGA) health authorities were contacted and their consent obtained before the actual work began. Furthermore, the local Ezes (traditional rulers of Ndiigbo), chiefs, and leaders of town development unions were briefed about the project, and their cooperation was sought in the mobilization of their people. During the parasitological and clinical surveys, health personnel from the LGA were always present to monitor safety standards.

### Census and mapping

All individuals in the selected communities who were more than one year of age were included in the study population, which comprised natives as well as non-natives who had resided there for at least one year. The target population was 1000 persons. During the census, the house registration numbers written on houses by the National Population Commission during the 1993 national census were used. Where such numbers were unavailable, one was provided. The appropriate positions of houses, markets, religious places, major roads and some track roads, as well as water bodies in the communities were noted.

### Skin snipping

Two skin snips (one from the shoulder and one from the waist) were taken for parasitological examination from each individual during daytime using a Walser corneo-scleral punch. The size of the biopsies was known to average 0.8 mg., with a range of 0.4 – 1.2 mg (14). The biopsies were placed in micro-titer wells containing 0.2 ml of 0.85% saline solution. When completed, each plate was covered with cellophane tape and taken to the laboratory where it was kept for 24 hours at room temperature (15). At the end of the 24-hour incubation period, the skin biopsies were fixed in formalin solution (35% formaldehyde solution) by adding two drops per micro-well. This was adapted from earlier studies (16–18). Thereafter, emerged microfilariae were observed and counted microscopically using ×40 magnification. Verification of the microfilariae as *O. volvulus* had been carried out in an earlier study by staining with Giemsa (19).

### Data analysis

The Epi-Info version 6.0 was used in entering data from parasitological survey, and SPSS for windows (1995 version) was used for data analysis. The geometric mean intensity (GMI) of microfilariae was calculated as antilog (Σlog (x+1)/n), with x being the number of mf per ml of blood in microfilaraemic individuals and n the number of microfilaraemic individuals examined.

## Results

An individual was considered mf positive if either of the waist or shoulder snips or both were mf positive. The mf intensities presented are averages of the two snips taken from each individual. The survey coverage was high (91.8% of the study population; 93.0% for males and 96.6% for females).

**Fig. 1:**
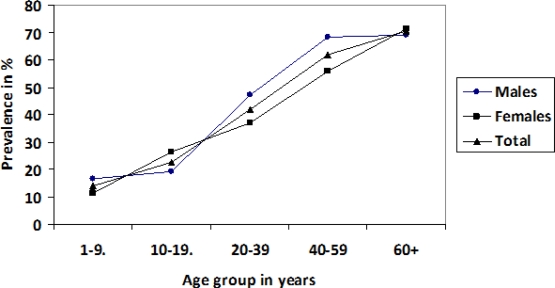
Prevalence of *Onchocerca volvulus* microfilaria in relation to age and sex

Thirty-seven percentage of those examined were positive for *O. volvulus* microfilariae (39.2% of males and 34.9% of females) and microfilariae appeared early in life. The mf prevalence in relation to age and sex is presented in Fig. 1. The youngest mf positive boy and girl were both four years old. In the youngest age group, 13.9% were positive for microfilaraemia. The mf prevalence increased steadily with increasing age to reach 70.4% in the oldest age group (69.2% for males and 71.4% for females). There was no significant difference in overall mf prevalence between males and females (*χ* ^*2*^-test; *P*>0.05). However, in the age groups 20–39 and 40–59 years, males had significantly higher mf prevalences than females (*χ* ^*2*^-test; *P*<0.05 for both tests).

The overall mf GMI among mf positive individuals was 16 mf/skin snip (18 mf/skin snip for males and 14 mf/skin snip for females). Males had higher mf GMI in all age groups except in the 10–19 years age group where it was the same in both sexes. The overall difference in mf GMI between males and females was statistically significant (*t*-test; *P*<0.01). The mf GMI in the first three age groups was comparable. The mf GMI rose significantly from 20–39 to the 40–59 years age group, and also from the 40–59 to the 60+ years age group, to peak at 35 mf/skin snip in the latter (*t*-test; *P*<0.001 for both tests). The highest individual mf intensity observed was 127 mf/skin snip (119 mf/skin snip from the waist snip and 134 mf/skin snip from the shoulder snip) in a 40 year-old man.

Comparison of microfilariae number of skin snips from the waist and the shoulder of the same individual showed that all who were positive in the shoulder skin snip were also positive in the waist skin snip. Six people were positive in the waist snip but negative in the shoulder snip. The mf GMI of those who were positive in both the waist and shoulder snippings (GMI=16 mf/skin snip, ranging from 1 to 127 mf/skin snip) exceeded significantly the mf GMI of those who were positive only in the waist snippings (GMI=2 mf/skin snip, ranging from 1 to 2 mf/skin snip).

A scatter plot of microfilariae numbers in snips from the waist against numbers in snips from the shoulder of the same individuals, showed that the mf counts from the waist and those from the shoulder were closely correlated (Pearson's correlation coefficient=+0.90; *P*<0.01). Expectedly, there was a more spread-out scattering of the low mf intensities, and furthermore, those with mf intensities below 10 mf/snip had a more scattering tendency away from the regression line than those with higher mf intensities.

## Discussion

The Upper Imo River Basin is traversed by rivers and several streams, and conditions are favorable for transmission of onchocerciasis. Eleven species of *Simulium*, including *S. damnosum s.l.* have thus been reported from the area (9). Since microfilarial density is important in on-set of clinical manifestations (8), the high prevalence of onchocerciasis-related skin manifestations reported in the UIRB (20), may be indicative of higher prevalence than reported here. This area should probably be regarded as the most severe onchocerciasis focus in the eastern part of Nigeria, forming a continuum of high onchocerciasis Endemicity with the Udi Hill range, Oji River Basin and the Anambra River Basin. The prevalence here is higher than reported in other areas in the region such as Ezeagu, Oji River and Uzo-Uwani areas of Enugu State (13), Igwun River Basin (21) and the Niger Delta (8). In the Lower Imo River Basin (LIRB), however, onchocerciasis was found only sporadically, and most infected were immigrants from endemic areas (22). It is worth noting that the skin-dwelling microfilaria of *Mansonella streptocerca* was not found in the Imo River Basin in this study, nor was it found in the neighboring areas of southeastern Nigeria where skin-snip examinations have been carried out for microfilariae^8^. This is contrary to the stipulations of Sasa (23) that *M. streptocerca* could be higher in the rainforest Nigeria than in the northern Savannah areas. On the contrary, *M. streptocerca* has been reported mostly in the Savannah parts of Nigeria (24, 25), and among Lagos residents (26), a cosmopolitan town, and home to Nigerians from several parts.

The mf prevalence increased steadily with age. This agrees with findings in Nigeria (8, 17, 27–29). This may be because older individuals have been exposed throughout their lives and that they are more exposed to the vectors because of their occupations, mostly as farmers in the fields, as opposed to the children who are attending school. The peak in biting in the late morning hours in the IURB (19), thus coincides with adults being in their fields while the children are in school. First vector contact occurs when children visit streams to swim, to fetch water for domestic use or for other purposes. Later in life, exposure continues during farming and other adult occupations. Resistance development is limited and the age-related prevalence and intensity therefore shows a gradual increase. In the highly endemic UIRB, there is frequent exposure to infective vectors from early childhood, and consequently infection is contracted early in life.

The prevalence of microfilaria was not significantly different between the sexes. This is in accordance with the results from the neighboring mesoendemic areas of Ezeagu and Oji-River Basin and Uzo-Uwani in Enugu State (13), and Bali District communities of Taraba State in northern Nigeria (30). It does not agree, however, with the findings of Anosike and Onwuliri (29) in Bauchi State, an area that is predominantly Moslem where traditional restrictions imposed on the women result in less exposure and thereby in lower prevalences than in men.

The overall GMI of 16 mf/skin snip among mf positive individuals in the UIRB study population may be regarded as low when compared with the mean microfilarial density of 50 mf per skin snip in Ezeagu, Orji-River, and Uzo-Uwani areas (13), and 169.2 mf/skin snip and 67.2 mf/skin snip observed in different areas of the Taraba River Valley (30, 31). This may be due to ivermectin treatment in the area carried out by the Nigerian Onchocerciasis Control Project (NOCP), which expectedly resulted in a drastic reduction in the microfilarial density and possibly reduction in the prevalence of microfilariae (32–36). This argument is corroborated by the results in the LIRB where there was no ivermectin treatment, and onchocerciasis is only sporadic, the overall mean mf intensity was 22 mf/skin snip (21). Furthermore, studies have shown that after treatment with invermectin, microfilariae would be reduced by half after 24 hours, by 85% after 72 hours, by 94% after one week, and by 98–99% after one to two months (37). Multiple treatments with ivermectin have marked effects on embryogenesis (38). Quantitative estimates have ranged from an irreversible decline in microfilarial production of approximately 30% per treatment (39), a reduction in the productivity index of 83% (40) to arrest of development at the single cell stage (41). Thus, when the factors listed above are taken into account, the presence of normal reproductive activity in female worms exposed to multiple treatments is incompatible with a normal response (42). However, there could be significant microfilaridermias despite multiple treatments with ivermectin and is mainly attributable to the non-response of the adult female worms and not to inadequate drug exposure or other factors (42). This was confirmed in a 30-month follow up study (43). Microfilariae in general remained sensitive to Ivermectin (42, 44).

The mf intensity was equal among the males than among females. This is consistent with the observations on another filarial species, *Wuchereria bancrofti* (45) that the microfilaria intensity is lower among women of reproductive age than among males of the same age.

Although the skin snips from the waist had a higher sensitivity for detecting microfilaria than skin snips from the shoulder, the difference presumably does not reflect a site preference. Thus, those who were positive in both the waist and shoulder skin snippings had a relatively higher GMI (16 mf/skin snip) than those who were positive only for the waist skin snip. A low microfilaria level may lead to a higher level of negatives in skin snipping using corneo-scleral punch, just as low microfilaria levels may lead to more false negatives when using thick smears in detection of *W. bancrofti* microfilariae (46). This is buttressed by the scatter-plot of the skin snip results from the waist and shoulder. This showed that those with mf intensities below 10 mf/snip had a more scattering tendency away from the regression line than those with higher intensities. Thus, there was a better agreement between results from the two skin snip sites when the mf intensities increased. The result was the same in the LIRB (19). Perhaps, 10 mf/snip is the critical threshold for parasitological survey methods involving corneo-scleral punch. This means that the method is not ideal for populations with low mf intensities.
